# Controlling Nutritional Status (CONUT) score is a prognostic marker in III-IV NSCLC patients receiving first-line chemotherapy

**DOI:** 10.1186/s12885-023-10682-z

**Published:** 2023-03-09

**Authors:** Yi Zhang, Fei-Fei Kong, Zheng-Qiu Zhu, Hai-Xia Shan

**Affiliations:** 1 Department of Oncology, the Suqian Clinical College of Xuzhou Medical University, Suqian, 223800 Jiangsu China; 2 Department of Oncology, the affiliated hospital of Xuzhou Medical University, Jiangsu, 221000 Xuzhou China

**Keywords:** Non‑small-cell lung cancer, Prognostic factor, CONUT score, PNI, SII, Chemotherapy

## Abstract

**Background:**

To investigate the prognostic impact of the controlling nutritional status (CONUT) score in non-small-cell lung cancer (NSCLC) patients receiving first-line chemotherapy.

**Methods:**

We retrospectively reviewed 278 consecutive patients undergoing chemotherapy for stage III-IV NSCLC between May 2012 and July 2020. CONUT score was calculated by incorporating serum albumin, total cholesterol, and total lymphocyte count. The patients were divided into two groups: CONUT ≥ 3 and CONUT < 3, according to receiver operating characteristic (ROC) analysis. The associations of CONUT with clinicopathological factors and survival were evaluated.

**Results:**

A high CONUT score was significantly associated with older age(*P* = 0.003), worse ECOG-PS(*P* = 0.018), advanced clinical stage(*P* = 0.006), higher systematic inflammation index (SII) (*P* < 0.001)and lower prognostic nutritional index (PNI) (*P* < 0.001).The high CONUT group had a significantly shorter progression-free survival(PFS) and overall survival(OS) than the low CONUT group. In the univariate analysis, higher SII, higher CONUT, advanced clinical stage and lower PNI were associated with worse PFS (*P*_all_ < 0.05). Worse ECOG-PS, higher SII, higher CONUT, advanced clinical stage and lower PNI were associated with worse OS (*P*_all_ < 0.05). In multivariate analysis, CONUT(HR, 2.487; 95%CI 1.818 ~ 3.403; *P* < 0.001) was independently associated with PFS, while PNI(HR, 0.676; 95%CI 0.494 ~ 0.927; *P* = 0.015) and CONUT(HR, 2.186; 95%CI 1.591 ~ 3.002; *P* < 0.001)were independently associated with OS. In ROC analysis, CONUT had a higher area under the ROC curve (AUC) for the prediction of 24-month PFS and OS than the SII or PNI. When the time-dependent AUC curve was used to predict PFS and OS, CONUT tended to maintain its predictive accuracy for long-term prognosis at a significantly higher level for an extended period after chemotherapy when compared with the other markers tested. The CONUT score showed better accuracy of predicting OS (C-index: 0.711) and PFS(C-index: 0.753).

**Conclusion:**

CONUT score is an independent prognostic indicator of poor outcomes for patients with stage III-IV NSCLC and is superior to the SII and PNI in terms of prognostic ability.

## Background

Non-small-cell lung cancer (NSCLC) is the most common cause of cancer-related death and a major public health problem worldwide, accounting for more than one million deaths annually [1, 2]. Over the last 20 years, the treatment strategies for advanced and metastatic NSCLC have dramatically changed. Although the treatments in lung cancer have made great progress [3], more effective treatment strategies must consider patient selection and evaluate the prognosis of patients with NSCLC. Recently, there has been increasing interest in identifying prognostic factors for tailored treatment.

Recently, patient immunonutritional status has been linked to treatment outcome. Immunonutritional status is an important feature of the tumour microenvironment and is associated with poor prognosis of various types of tumours [4, 5]. Prognostic nutritional index (PNI) is a proven scoring system based on immunonutritional status that allows an estimation of treatment tolerability and cancer progression [6]. Inflammation is also reported to be associated with cancer prognosis. Haematological inflammatory parameters such as neutrophils, lymphocytes, monocytes, and platelets can reflect systemic and focal inflammation and have important value in predicting the prognosis of tumours, including NSCLC [7, 8].

The Controlling Nutritional Status (CONUT) score is a scoring system for patient immunonutritional status which has attracted substantial attention and is reported to be associated with clinical outcomes in various malignancies. Similar to the PNI and systematic inflammation index (SII) [9, 10],the CONUT score is easily obtained and calculated from three clinical parameters: serum albumin (protein reserve), total cholesterol (caloric depletion), and total lymphocyte count (immune defense) [11]. Therefore, CONUT score represents three important immunonutritional indices, which was was first proposed by Ignacio et al. [12]. It has been reported that the CONUT score can be a predictive or prognostic marker in many types of cancers. For NSCLC, some reports have shown that the CONUT score is useful for predicting long-term outcomes of surgery and immune therapy [13–15]. However, few studies have reported whether the CONUT score is associated with the prognosis of NSCLC patients treated with chemotherapy.

The aim of this retrospective study was to determine whether prechemotherapy CONUT score could be a useful predictor of survival in patients with NSCLC and to compare the accuracy of the CONUT score, PNI and SII as predictors of the survival rate of such patients.

## Methods

### Patients

We retrospectively enrolled 278 patients diagnosed with advanced NSCLC who regularly received DP (docetaxel plus cisplatin), GP (gemcitabine plus cisplatin), NP (vinorelbine plus cisplatin), PC (pemetrexed plus cisplatin) and TP (paclitaxel plus cisplatin) chemotherapy regimens at the Affiliated Hospital of Xu Zhou Medical University from January May 2012 and July 2020.

The inclusion criteria were as follows: (1) NSCLC was pathologically diagnosed; (2) NSCLC was stage III or IV according to the American Joint Committee on Cancer (AJCC) 8th edition; (3) the patient received chemotherapy for more than two cycles without a combination of targeted therapy, radiation therapy and immune therapy; (4) the patient had no other cancer history and laboratory test results were obtained before treatment.

The exclusion criteria were as follows: (1) patients with missing or incomplete data; (2) patients who underwent surgery, radiotherapy, immunotherapy before standard chemotherapy protocols, (3) patients who had obvious fever and pneumonia before chemotherapy.

This retrospective study was approved by the ethics committee of the Affiliated Hospital of Xu Zhou Medical University.

### Data collection and follow-up

Data, including age, sex, histological subtype, stage, smoking status, chemotherapy regime, Eastern Cooperative Oncology Group Performance Status (ECOG PS) scores, routine blood parameters and biochemical profiles, were collected retrospectively from individual medical case notes, electronic patient records and pathology reports. Blood samples were obtained and assayed within 2 weeks before chemotherapy. CONUT scores were summarized using the serum albumin concentration, peripheral lymphocyte counts and the total cholesterol concentration, as described in Table 1. The following formula was used to calculate PNI and SII. PNI: albumin (g/L) × total lymphocyte count × 10^9^/L. SII: platelet count × neutrophil count/lymphocyte count [9, 16]. Follow-up was performed every 3 months. All patients were monitored either until July 2020 or until death. The median follow-up time was 24 months (range, 3–75 months). Progression-free survival (PFS) was defined as the interval from treatment initiation until disease progression or death. Overall survival (OS) was defined as the interval from treatment initiation until the date of death or the date of last follow-up for patients who had not died before the censor date.

**Table 1:** The CONUT scoring system

Parameters	Degree of undernutrition			
	**Normal**	**Light**	**Moderate**	**Severe**
Serum albumin (g/dL)	≥ 3.5	3.0–3.4	2.5–2.9	< 2.5
score	0	2	4	6
Total lymphocyte count (mm^3^)	≥ 1600	1200–1599	800–1199	< 800
score	0	1	2	3
Total cholesterol (mg/dl)	≥ 180	140–179	100–139	< 100
score	0	1	2	3
CONUT score (total)	0–1	2–4	5–8	9–12

*CONUT* Controlling nutritional status

## Statistical analysis

The patients were classified into two groups based on receiver operating characteristic (ROC) curves. We obtained optimal cut-off values of CONUT, SII and PNI via calculating best Youden index. The associations between CONUT score and clinicopathological characteristics were analysed using χ2 tests. A comparison of the time-dependent AUC-of-ROC curves and Harrell’s concordance index (C-index) for the prediction of PFS and OS was performed to seek more superior biomarker. Survival analysis was performed using Kaplan–Meier method. The differences between the survival curves were compared by log-rank test. Cox proportional hazards regression models were used to calculate hazard ratios (HRs) and 95% confidence intervals (CIs). A *P* value < 0.05 was considered to indicate statistical significance. The results were analysed using SPSS 21.0.

## Results

### Baseline characteristics of patients

In total, 278 cases were enrolled in the present study, of which 192 (69.1%) patients were male, 162 (58.3%) patients had a smoking history, and 176 (63.3%) patients had stage IV disease. Based on the ROC curve to predict 24-month overall survival (OS), the best cut-off value was 3 for CONUT, 984.72 for SII and 48.95 for PNI. Therefore, a total of 114 (41.0%) patients were classified into the CONUT-high(≥ 3) group, and 164 (59.0%) patients were classified into the CONUT-low(< 3) group. Compared with CONUT-high group, the nutritional status of CONUT-low group was better. The characteristics of all patients are detailed in Table 2. A high CONUT score was significantly associated with older age(*P* = 0.003), worse ECOG PS(*P* = 0.018), advanced clinical stage(*P* = 0.006),higher SII (*P* < 0.001)and lower PNI (*P* < 0.001).

**Table 2:** The relationship between CONUT score and clinicopathological characteristics of the patients

Variable	Total(*n* = 278)	CONUT score		*χ*^*2*^	*P*
		< 3(***n*** = 164)	≥ 3(***n*** = 114)		
Gender				0.742	0.389
Male	192(69.1%)	110(67.1%)	82(71.9%)		
Female	86(30.9%)	54(32.9%)	32(28.1%)		
Age				8.537	0.003
< 60	109(39.2%)	76(46.3%)	33(28.9%)		
≥ 60	169(60.8%)	88(53.7%)	81(71.1%)		
Smoking status				1.276	0.259
Minimal/never	116(41.7%)	73(44.5%)	43(37.7%)		
Current/former	162(58.3%)	91(55.5%)	71(62.3%)		
ECOG-PS				5.623	0.018
0/1	160(57.6%)	104(63.4%)	56(49.1%)		
2	118(42.4%)	60(36.6%)	58(50.9%)		
Histology subtype				3.272	0.195
Squamous	113(40.6%)	60(36.6%)	53(46.5%)		
Adenocarcinoma	133(47.8%)	82(50.0%)	51(44.7%)		
others	32(11.5%)	22(13.4%)	10(8.8%)		
TNM staging				7.504	0.006
III	102(36.7%)	71(43.3%)	31(27.2%)		
IV	176(63.3%)	93(56.7%)	83(72.8%)		
Chemotherapy regimens				5.691	0.233
DP	32(11.5%)	17(10.4%)	15(13.2%)		
GP	97(34.9%)	55(33.5%)	42(36.8%)		
NP	37(13.3%)	23(14.0%)	14(12.3%)		
PC	83(29.9%)	56(34.1%)	27(23.7%)		
TP	29(10.4%)	13(7.9%)	16(14.0%)		
CEA				0.764	0.382
Normal	123(44.2%)	69(42.1%)	54(47.4%)		
High	155(55.8%)	95(57.9%)	60(52.6%)		
CYF				0.750	0.386
Normal	76(27.3%)	48(29.3%)	28(24.6%)		
High	202(72.7%)	116(70.7%)	86(75.4%)		
NSE				0.638	0.424
Normal	153(55.0%)	87(53.0%)	66(57.9%)		
High	125(45.0%)	77(47.0%)	48(42.1%)		
SII				30.353	0.000
< 984.72	140(50%)	60(37%)	80(70%)		
≥ 984.72	138(50%)	104(63%)	34(30%)		
PNI				53.448	0.000
≥ 48.95	129(46%)	106(64.0%)	23(20%)		
< 48.95	149(54%)	58(36.0%)	91(80%)		

*ECOG-PS * Eastern Cooperative Oncology Group performance status, *TNM * Tumor-node-metastasis, *CONUT * Controlling nutritional status, *SII * Systemic immune-inflammation index, *PNI * Prognostic nutritional index, *DP * Docetaxel plus cisplatin, *GP * Gemcitabine plus cisplatin, *NP * Vinorelbine plus cisplatin, *PC * Pemetrexed plus cisplatin, *TP * Paclitaxel plus cisplatin, *CEA * Carcinoembryonic antigen, *NSE * Neuron-specific enolase, *CYF * Cytokeratin-19-fragment

### Comparison of CONUT with other prognostic factors (SII and PNI) in terms of prognostic accuracy

**Fig. 1:**
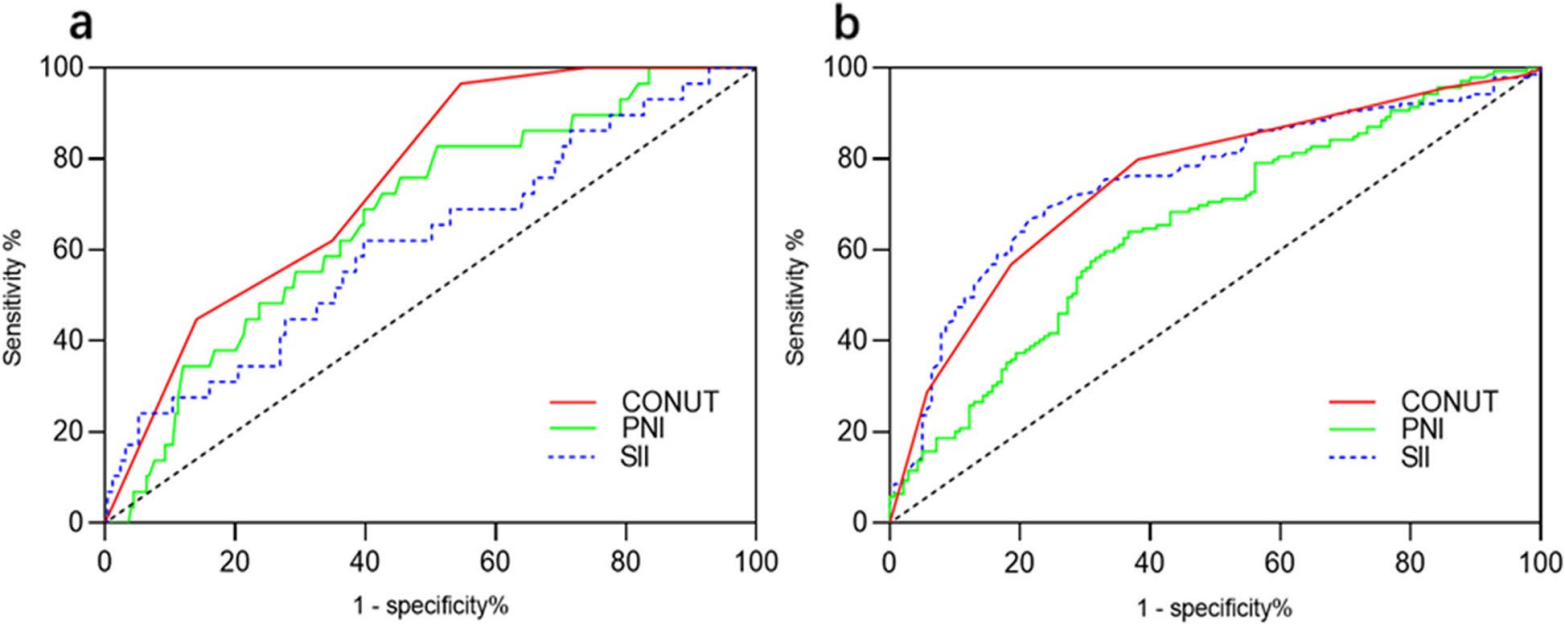
The ROC curves of CONUT, SII and PNI for predicting 24-month PFS (**a**) and OS (**b**) CONUT, controlling nutritional status; SII, systemic immune-inflammation index; PNI, prognostic nutritional index

Using the 24-month survival as an endpoint, 3 was considered to be the best cut-off value for CONUT since the corresponding Youden index was maximal. The sensitivity and specificity for OS were 79.9% and of 61.9%, respectively (Fig. 1a,b). All the patients were classified into were divided into two groups: CONUT ≥ 3 and CONUT < 3. The AUCs of SII, PNI, and CONUT for 24-month PFS were 0.616 (95% CI: 0.504–0.727), 0.676(95% CI: 0.580–0.771), and 0.750(95% CI: 0.672–0.827), respectively (Fig. 1a), while the AUCs of SII, PNI and CONUT for 24-month OS were 0.653(95% CI: 0.589–0.717), 0.753(95% CI: 0.695–0.811), and 0.753(95% CI: 0.695–0.810), respectively (Fig. 1b). CONUT showed significantly higher accuracy than SII and PNI in the prediction of 24-month PFS. CONUT showed significantly higher accuracy than SII in the prediction of 24-month OS. However, the predictive accuracy of CONUT was same to PNI in relation to 24-month OS. The C-index of CONUT.for OS and PFS was 0.711 and 0.753. The C-index of SII for OS and PFS was 0.465 and 0.469.

The C-index of PNI for OS and PFS was 0.562 and 0.696.

**Fig. 2:**
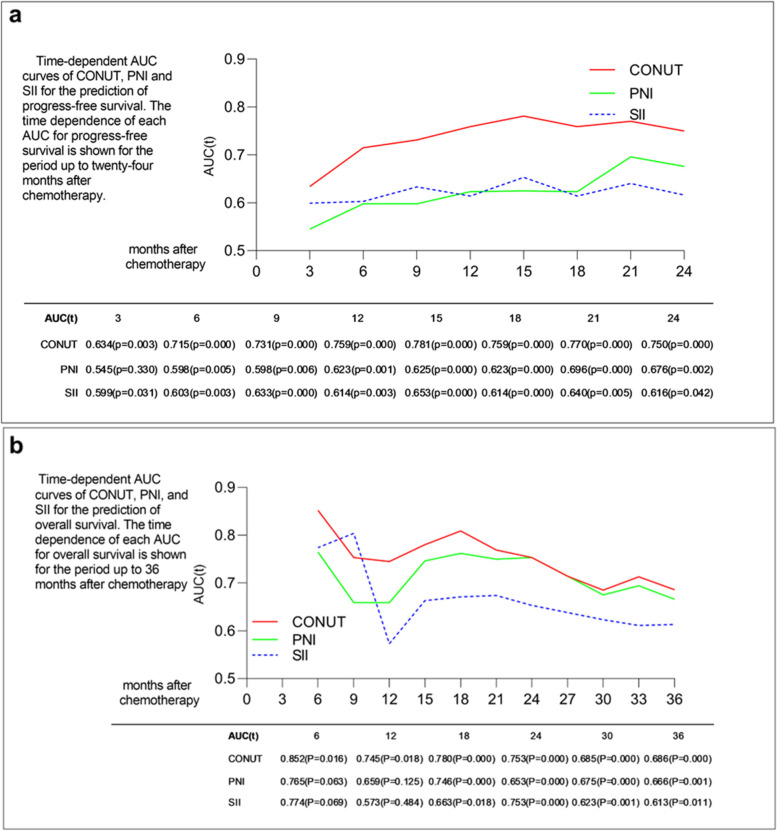
Time-dependent AUC-of-ROC curves for the prediction of PFS (**a**) and OS (**b**)

A comparison of the time-dependent AUC-of-ROC curves for the prediction of PFS (Fig. 2a) and OS (Fig. 2b) was performed. The AUC of CONUT for PFS tended to be higher than the other scoring systems at all time points tested. The AUC of CONUT tended to be higher than the other scoring systems at time points tested except the prediction of the 9-month and 24-month OS.

A comparison of the time-dependent AUC-of-ROC curves for the prediction of PFS (Fig. 2a) and OS (Fig. 2b) was performed. The AUC of CONUT for PFS tended to be higher than the other scoring systems at all time points tested. The AUC of CONUT tended to be higher than the other scoring systems at time points tested except the prediction of the 9-month and 24-month OS.

### Prognostic value of SII, PNI, and CONUT

**Fig. 3:**
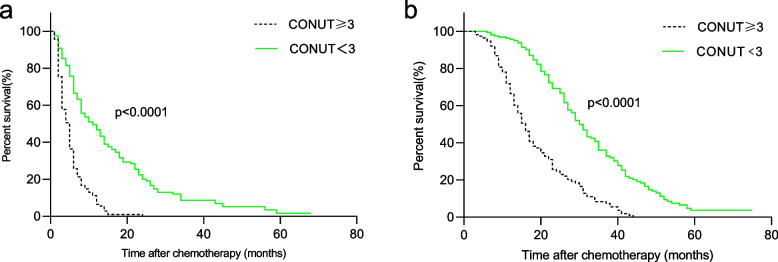
Kaplan–Meier survival analyses of the correlation between CONUT and survival among NSCLC patients: PFS (**a**) and OS (**b**). CONUT, controlling nutritional status

**Fig. 4:**
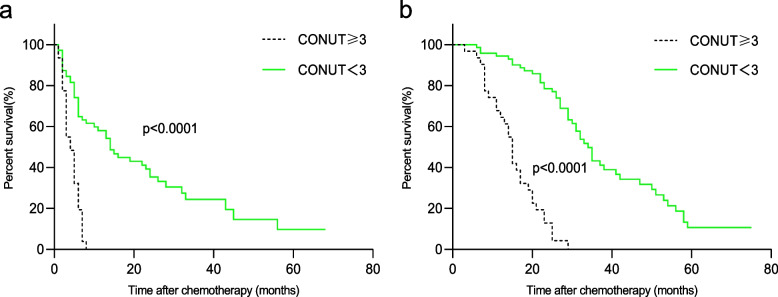
Kaplan–Meier survival analyses of PFS (a) and OS (b), according to CONUT, among patients in the stage III subgroup. CONUT, controlling nutritional status

**Fig. 5:**
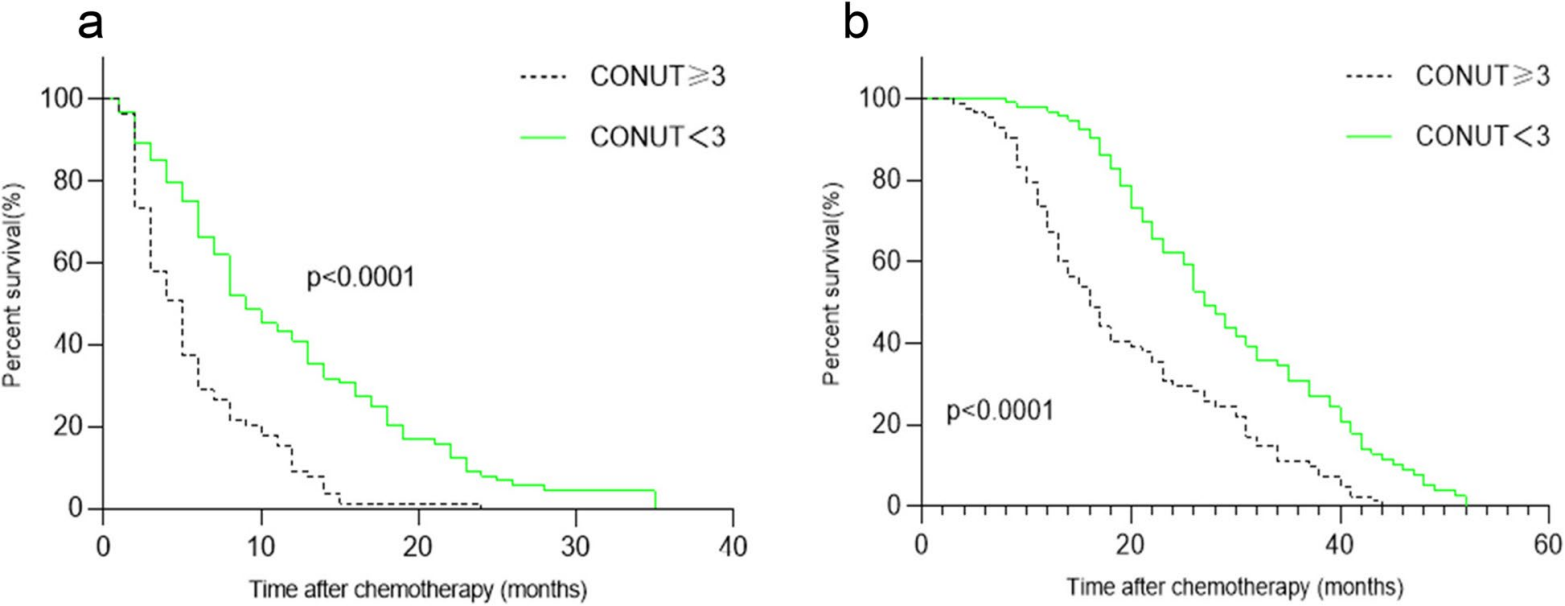
Kaplan–Meier survival analyses of PFS (**a**) and OS (**b**), according to CONUT, among patients in the stage IV subgroup. CONUT, controlling nutritional status

In the present study, we found that CONUT < 3 before treatment was associated with longer PFS and OS (Fig. 3). After stratification by TNM stage, the prognostic significance of the CONUT score was also maintained in patients with stage III (Fig. 4) and stage IV (Fig. 5) disease.

A univariate analysis of the factors associated with PFS indicated that higher SII, higher CONUT, advanced clinical stage and lower PNI were factors associated with worse survival (Table 3). A multivariate analysis indicated that CONUT was significant independent prognostic parameters for PFS (Table 4).

**Table 3:** Univariate analysis of potential factors associated with PFS and OS [Median (Q25, Q75)]

Variable	Total(n = 278)	PFS	*χ*^*2*^	*P*	OS	*χ*^*2*^	*P*
Gender			0.769	0.381		2.584	0.108
Male	192(69.1%)	6.0(5.16,6.85)			25.0(22.25,27.75)		
Female	86(30.9%)	6.0(4.18,7.82)			26.00(20.88,31.12)		
Age			0.899	0.343		1.881	0.170
< 60	109(39.2%)	6.0(5.28,6.72)			26.0(22.96,29.05)		
≥ 60	169(60.8%)	6.0(4.84,7.16)			23.0(19.26,26.74)		
Smoking status			0.065	0.799		1.598	0.206
Minimal/never	116(41.7%)	6.0(4.76,7.24)			25.0(20.25,29.75)		
Current/former	162(58.3%)	6.0(4.96,7.04)			25.0(22.16,27.84)		
ECOG-PS			1.615	0.204		5.642	0.018
0/1	160(57.6%)	6.0(4.97,7.03)			27.0(24.68,29.32)		
2	118(42.4%)	6.0(4.94,7.06)			22.0(19.76,24.24)		
Histology subtype			0.646	0.724		1.730	0.421
Squamous	113(40.6%)	6.0(4.85,7.15)			25.0(22.48,27.52)		
Adenocarcinoma	133(47.8%)	6.0(4.67,7.33)			25.0(20.72,29.28)		
others	32(11.5%)	6.0(4.63,7.38)			26.0(15.7,36.26)		
TNM staging			6.549	0.010		12.981	0.000
III	102(36.7%)	6.0(5.24,6.76)			27.0(23.35,30.65)		
IV	176(63.3%)	6.0(4.76,7.24)			23.0(20.23,25.77)		
Chemotherapy regimens			1.156	0.885		1.419	0.841
DP	32(11.5%)	5.0(1.67,8.33)			20.0(14.46,25.54)		
GP	97(34.9%)	6.0(4.79,7.21)			26.0(22.08,29.92)		
NP	37(13.3%)	7.0(4.02,9.98)			23.0(15.87,30.13)		
PC	83(29.9%)	6.0(5.01,6.99)			25.0(20.71,29.29)		
TP	29(10.4%)	5.0(3.49,6.51)			26.0(19.87,32.13)		
CEA			3.240	0.072		2.163	0.141
Normal	123(44.2%)	6.0(5.06,6.94)			25.0(22.02,27.98)		
High	155(55.8%)	6.0(4.54,7.46)			26.0(22.39,29.61)		
CYF			3.147	0.076		1.105	0.293
Normal	76(27.3%)	6.0(4.88,7.13)			25.0(21.92,28.08)		
High	202(72.7%)	6.0(4.97,7.03)			25.0(22.08,27.93)		
NSE			0.945	0.331		1.406	0.236
Normal	153(55.0%)	6.0(4.99,7.01)			23.0(20.49,25.51)		
High	125(45.0%)	6.0(4.71,7.29)			26.0(22.07,29.93)		
SII			9.309	0.002		25.937	0.000
< 984.72	140(50%)	7(5.45,8.54)			30.0(27.25,32.75)		
≥ 984.72	138(50%)	5(4.26,5.74)			18.0(15.58,20.42)		
PNI			13.631	0.000		48.178	0.000
≥ 48.95	129(46%)	7.0(5.81,8.19)			31.0(27.69,34.31)		
< 48.95	149(54%)	5.0(4.15,5.86)			18.0(15.51,20.49)		
CONUT score			62.912	0.000		78.963	0.000
< 3	164(59.0%)	8.0(5.72,10.28)			30.0(27.58,32.42)		
≥ 3	114(41.1%)	4.0(3.13,4.87)			16.0(14.17,17.83)		

*ECOG-PS * Eastern Cooperative Oncology Group performance status, *TNM * Tumor-node-metastasis, *CONUT * Controlling nutritional status, *SII * Systemic immune-inflammation index, *PNI * Prognostic nutritional index, *DP * Docetaxel plus cisplatin, *GP * Gemcitabine plus cisplatin, *NP * Vinorelbine plus cisplatin, *PC * Pemetrexed plus cisplatin, *TP * Paclitaxel plus cisplatin, *CEA * Carcinoembryonic antigen, *NSE * Neuron-specific enolase, *CYF * Cytokeratin-19-fragment

**Table 4:** Results of the multivariate Cox regression analysis of factors predicting the PFS and OS

**Variable**	**PFS**			**OS**		
	***HR(95% CI)***	***Waldχ***^***2***^	***P***	***HR(95% CI)***	***Waldχ***^***2***^	***P***
SII	1.109(0.847 ~ 1.452)	0.565	0.452	1.288(0.973 ~ 1.704)	3.133	0.077
PNI	0.965(0.718 ~ 1.297)	0.056	0.812	0.676(0.494 ~ 0.927)	5.931	0.015
CONUT score	2.487(1.818 ~ 3.403)	32.448	0.000	2.186(1.591 ~ 3.002)	23.307	0.000
ECOG-PS	-	-	-	1.020(0.786 ~ 1.324)	0.22	0.882
TNM staging	1.077(0.818 ~ 1.420)	0.281	0.596	1.203(0.891 ~ 1.624)	1.451	0.228

In Cox hazard analyses, univariate analysis showed that ECOG-PS, SII, CONUT, clinical stage and PNI were significantly associated with OS (Table 3). After the exclusion of variables that showed no impact on OS in univariate analysis, Cox multivariate regression analysis was performed, which identified only PNI and CONUT as independent prognostic factors of OS (Table 4).

## Discussion

In the present study, we demonstrated the prognostic value of CONUT in III-IV NSCLC. To our knowledge, this is the first report investigating the prognostic value of CONUT and comparing the superiority between nutrition-based indices and inflammation-based indices in patients with advanced NSCLC treated with chemotherapy. Moreover, the results indicated that CONUT score was associated with age, ECOG-PS, clinical stage, SII and PNI. Significantly, without considering the tumour stage, CONUT independently predicted the prognosis of NSCLC patients. Compared with low CONUT scores, high CONUT scores predicted shorter PFS and OS. Similarly, Gul has also reported that high CONUT score is associated with poor prognosis in patients with locally advanced and advanced stage lung cancer. Patients should be screened for nutritional status and supported [17]. However, in our study, the nutritional score is further compared with the inflammation indicator.

As mentioned above, the prognostic nutritional index, as a nutrition-based index, which was calculated from the serum albumin concentration and the total peripheral lymphocyte count, has been reported to associate with survival in NSCLC patients [18]. It is not difficult to see that the two indices of PNI are covered by the CONUT scoring system. The serum albumin concentration is a common nutritional status indicator that can be influenced by many other factors, such as liver function, inflammation, infection, dehydration and so on [12, 19]. Hence, to reduce the weight of serum albumin, some scholars proposed adding plasma cholesterol levels to optimize the PNI scoring system [20]. In addition, cytokines and CRP also modulate the production of albumin [12, 19]. As cholesterol plays a crucial role in influencing cell membrane fluidity, cholesterol affects the mobility of cell surface receptors and their ability to transmit signals. Moreover, serum cholesterol levels influence caloric intake [20]. Lymphocytes play a key role in initiating cellular immunity, and high numbers of infiltrating lymphocytes are associated with good prognosis [21, 22]. Therefore, the combination of these three parameters could balance the impact of each parameter.

Inflammation-based indices also act as oncological prognosis biomarkers. A series of inflammation indices, such as NLR, PLR, LMR and SII, showed positive correlations with poor survival outcome in patients with lung cancer [19, 23, 24]. Some reports have also illuminated that the SII is a superior prognostic factor for survival outcome compared to the NLR and PLR [25]; therefore, we selected the SII as the representative inflammation prognostic index. It is not hard to see that serum albumin and cholesterol are not only nutrition indices but also inflammatory indices. However, the SII is a pure inflammatory index based on neutrophils, lymphocytes, and platelets and is more easily affected by external factors, such as pneumonia. Although the AUC-of-ROC of CONUT is not higher than SII in the prediction of 9-month OS and similar to the prediction of 24-month OS, the overall level, CONUT was the best predictor of long-term survival in cases with NSCLC among the three indices analysed in the present study. Therefore, we think that CONUT is a superior prognostic biomarker that not only reflects the features of tumour cells but also reflects the nutritional status of patients.

The optimal cut-off values for PNI and SII remain undefined for relatively few studies have examined PNI and SII in patients with NSCLC. Due to heterogeneity among patents and low sample sizes, various values have been used in previous reports. In our study, the optimal cut-off value of CONUT, which was similar to most other previous articles, was 3. Therefore, compared with PNI and SII, CONUT is a more superior prognostic biomarker.

The present study has certain limitations. First, this is a retrospective analysis; hence, there are several potential factors that might have influenced the studied results, such as lipid-lowering agents. Second, data for all patients were collected from a single institute, and the number of patients was relatively small. Third, different nutritional support in the process of chemotherapy might have confounded our results. Therefore, a multi-institutional investigation, especially a prospective validation study, is needed to confirm the results.

## Conclusion

The present study indicates that CONUT is an independent prognostic indicator of poor outcomes for patients with stage III-IV NSCLC and is superior to SII and PNI in terms of prognostic ability.

## Data Availability

The datasets used and/or analysed during the current study are available from the corresponding author on reasonable request.

## Abbreviations

HR: Hazard ratio
95% CI: 95% Confidence interval
SII: Systemic immune inflammation index
PNI: Prognostic nutritional index
ECOG-PS: Eastern Cooperative Oncology Group performance status
CONUT: Controlling nutritional status
TNM: Tumor-node-metastasis
